# Patients’ lived experience and reflections on long COVID: an interpretive phenomenological analysis within an integrated adult primary care psychology NHS service

**DOI:** 10.1186/s41687-023-00570-2

**Published:** 2023-03-20

**Authors:** Lilian Skilbeck, Christopher Spanton, Michael Paton

**Affiliations:** East London NHS Foundation Trust, Newham Talking Therapies, Vicarage Lane Health Centre, 10 Vicarage Lane, Stratford, E15 4ES UK

**Keywords:** Long COVID, Lived experience, Physical symptoms, Mental health impacts, Self-management, Recovery

## Abstract

**Background:**

Long Covid is an unknown illness which has been shown to affect sufferers regardless of mild initial COVID-19 or age. There is still a lot unknown about long COVID illness. There has been a call to understand this illness not only from a professional standpoint but also through the lived experience of patients. Patient reported outcomes through lived experience research is one such angle. To date, no research has explored the overall lived experiences and long COVID illness trajectory perspectives of the patients. This study aimed to gather patient reported outcomes of their long COVID through lived experience research. It recruited adult participants aged 18-years and over who had been referred to a primary care integrated psychology service. The study employed qualitative research using semi-structured interviews and Interpretive Phenomenological Analysis methodology.

**Results:**

A total of eighteen participants completed the study. From their lived experiences, the participants uncovered the varied symptoms of long COVID. They also uncovered their lived progression of living with an unknown chronic illness. Common themes included uncertainty, mental and social impacts, and the processes of self-advocacy, mastering their symptoms, subjective recovery and future coping.

**Conclusion:**

This study uncovered the lived experience of long COVID in participants. The results from this study uncovered the lived subjective biopsychosocial experiences of long COVID chronic illness. Traditionally, patients receive care and recommendations from healthcare professionals. However, as long COVID is a new illness, this care model was limited. The participants in the current study described being left with a sense of uncertainty and role confusion. However, participants were able to realize their locus of control over their long COVID illness progression. This illustrates that patients have the resources to uncover the unknowns of this new illness which could inform clinical practice and further research. This suggests that that long COVID needs to be approached from a biopsychosocial perspective which emphasises patient involvement.

## Background

Coronavirus disease (COVID-19) is an infectious disease from the SARS-CoV-2 virus [1]. The COVID-19 pandemic has had a major impact on global health since its emergence in 2019. This disease remains poorly understood due to its recency and atypical presentations. One of these presentations is the persistent post-acute symptoms experienced by some people. In the UK, over 2.1 million people have reported symptoms persisting for more than four weeks as of 5 January 2023 [2]. These persistent symptoms have been coined long COVID and have emerged as a healthcare and social issue [3]. Therefore, there is a need to learn about long COVID and what it means for the health of the UK and worldwide population.

### What is long COVID?

Long COVID (also referred to as post-acute sequelae of SARS-CoV-2 infection, or PASC) is the collective term for symptoms lasting for four weeks or more post-acute Coronavirus infection. Research shows that some symptoms of long COVID resemble those of chronic fatigue syndrome or post viral fatigue syndrome from other infections such as influenza [4]. However, the difference is that long COVID has a whole array of additional new symptoms. Data from the National Institute for Health Research [5] suggests four symptom clusters: post-viral fatigue and/ or breathlessness; fluctuating multisystemic symptoms; organ damage; post-intensive care symptoms (chronic pain, cognitive impairment, mental health problems). Research shows that long COVID can affect both adults and children. There are currently more than two hundred long COVID symptoms have been identified to date [5]. Additionally, researchers found that both mild and severe cases of COVID-19 are associated with long COVID. However, there is research to suggest a correlation between the severity of long COVID symptoms and admission to hospital or intensive care unit (ICU). For example, post-COVID ICU patients have been shown to exhibit higher levels of physical health problems such as pain and organ damage. They have also been shown to exhibit more mental health problems including depression, anxiety and/ or ICU post-traumatic stress disorder [6, 7]. There is also research to suggest that the impacts of long COVID differ by age, gender and pre-existing health status [2]. In addition to the physical and psychological effects of long COVID, it also has social and economic consequences for the sufferers. For example, long COVID has impacts on daily activities, employment and social connections [8].

The National Institute for Health and Care Research [9] have acknowledged that the clusters of long COVID symptoms still remain misunderstood and that for some people, there appears to be potential for further deterioration. They suggested that long COVID care requires a multiprofessional workforce strategy. They also recommend that since long COVID is a multisystemic illness with more than two hundred symptoms, it affects people differently. They emphasise the need to investigate anyone with persistent symptoms, including non-hospitalised populations. A key recommendation is that as long COVID is a complex and atypical disease, research strategies from all angles are needed. Emerging evidence suggests that long COVID could become a global health burden [1]. This calls for research to understand it. There is a growing number of research studies on long COVID. However, most of the current research has been focused on the physiological impacts of the illness [10]. One of the key NIHR recommendations is the call for research involving the perspective of people with the experience of long COVID [5]. This is important as these are experts by experience and are in position to articulate lessons and suggestions from an insider’s perspective.

### Patient-led construction of long COVID

Chronic illnesses are persistent, with consequences including uncertainty, disability and decreased quality of life [11]. They often have a course that varies over time depending on the aetiology and physiology of the illness. Common challenges for sufferers include recognising symptoms, taking appropriate actions, working with healthcare professionals and effective management over time. This is especially due to the fact that illness progression does not appear to be linear. Long COVID is believed to be the first illness collectively constructed by patients [3]. In the early stages of this illness, patients experienced unexplained symptoms but often felt neglected and disbelieved. This led to patients seeking collective support on social media platforms. Some groups also commissioned their own research to use patient knowledge to make better sense of their experiences. This patient-led drive helped shape the current scientific understanding of long COVID. As acknowledged by several authors [12, 13], this highlights the need to continuously involve patients and incorporate their perspectives and knowledge alongside their illness trajectories.

### Clinical relevance of lived experience research

The experience of chronic illness is a complex and ever-changing process that that is often difficult to unravel [11]. Lived experience research in healthcare illuminates the perspectives and experiences of people who living with the phenomenon under study [14]. The patient has an experience which may be seen as a source of a knowledge about an illness. Lived experience research goes beyond the apparent obviousness of the illness and its conception as a self-contained world by revealing its biopsychosocial dimensions [15]. For example, lived experience research has been used in understanding conditions such as chronic pain [16]. However, the application of lived experience research in the UK National Health Service (NHS) is still limited. It is hoped that this may change given the current NHS patient and public involvement agenda [17]. The benefit of lived-experience research is that it can enhance methodological sensitivity, data accuracy, validity of results, and overall relevance to service users. Findings from lived experience research can also be used to inform future practice and research. For example, patients could be involved in co-production and co-design of treatments. They could also be involved in research as illustrated by the NIHR. Lived experience research also has the potential to benefit wider populations in their recovery journeys through learning from the insights, challenges and successes of others. This is particularly important for long COVID as it is a new illness with atypical symptoms and no current standardised treatments. Lived experience research participants have also reported benefits of involvement including making a difference, satisfaction empowerment and hope [14].

### Why is the current study necessary?

Although long-COVID is a new illness, biomedical researchers have drawn on other similar illnesses such as myalgic encephalomyelitis/chronic fatigue syndrome (ME/CFS) and dysautonomia to understand it [18]. However, there is a widespread lack of knowledge these similar viral-onset illnesses and long-COVID is a multisystem illness, Therefore, research on this disease is often not built on past findings. The current consensus is that the biopsychosocial characteristics and of long COVID are still not understood. For example, Siddaway [19] has suggested that a biopsychosocial perspective has much to offer in understanding long COVID, and that healthcare professionals have a key role to play. However, the role of the patient also needs to be emphasised. Lived experience research would help meet this need. The existing research on long COVID is largely experimental, survey-driven and descriptive in nature. To date, qualitative patient experience research has focused on patient narratives, content analysis, thematic analysis and grounded theory (e.g., [20, 21]). However, no qualitative research to date has explored the phenomenological lived experience and subjective patient reported illness trajectories of long COVID. Research also shows that patients demand their version of reality to be recognized [22]. Therefore, the current study aimed to fill this gap.

### Study aims and objectives

The aim of the current study was to obtain an in depth phenomenological understanding of the lived experience of long-COVID illness trajectory following COVID-19 illness.

The objectives were to: recruit participants from patients presenting to the integrated adult primary care service with various atypical long COVID symptoms; use the qualitative interview method and interpretive phenomenological analysis (IPA) to derive themes.

In line with the NHS England agenda for patient and public involvement [23], the research question, interview schedule and study protocol were actively reviewed by two independent service users with an experience of long COVID. This ensured that the research was fit-for-purpose and informed from the perspective of those with long COVID.

## Methods

### Procedure

This study employed qualitative research method where data were collected using semi-structured interviews and were analysed with an interpretative phenomenological analysis (IPA) methodology. This approach is founded on hermeneutic phenomenology, which aims to explore in depth individual perceptions of significant experiences [24]. This exploration is deep-rooted and subjective to the individual and can only be accessed by the researcher through interpretation in order to obtain an insider’s perspective. The idiographic nature of IPA allows for rigorous explorations of the meanings and reflections on the experience. This idiographic approach has been used in healthcare research as it helps elucidate how experiences affect patients which then informs intervention strategies [25].

Before commencing the study, ethical approval was obtained from North East-York Research Ethics Committee (National Health Service UK, Health Research Authority).

### Recruitment

Participants were recruited from patients who had attended the adult primary care integrated mental health service. Participants were eligible for the study if they were aged 18-years and over who had the experience of living with long COVID. Participants included patients at any stage of their treatment or at follow-up. Participants were made aware of the study by a flyer which was displayed in the communal rooms or provided with information by the clinician of contact. The researchers were not involved in the participants’ direct care. Potential participants who picked up the flyer voluntarily contacted the primary author using the contact details on the flyer. Six potential participants made contact. Those expressing an interest to the clinician of contact voluntarily gave their consent to be contacted by the primary author. Fifteen potential participants were contacted. Potential participants had a formal discussion with the researchers and were pre-screened against the study inclusion criteria. Those meeting the criteria and still wishing to take part were provided with a detailed participant information sheet on the study and consent form to return should they still wished to proceed. Participants were also informed that they could withdraw their consent at any time should they wish.

The informed consent form provided information on the purpose of the research, detailed potential risks, and a confidentiality statement of how participant information would be securely handled. It also provided information on any further support that may be required as a result of taking part. Participants were allowed 48 h to read the information and make their fully informed decision. Participants meeting the inclusion criteria and willing to take part returned their informed consent in writing. Those who did not return the form within this time were sent an email reminder once. Participants were then offered an interview date and time following their informed written consent. All participants included in the study had either received a diagnosis of long COVID or a recognition of post-COVID syndrome. The study sample comprised of a wide range of characteristics (age, gender, ethnicity and occupation). As shown in Table 1, this consisted of 18 participants (13 female and 5 male) who were anonymised and labelled PA to PR.

**Table 1:** Participant characteristics (n = 18)

ID	Gender	Age (years)	Ethnicity	Occupation	Time in months since acute infection	Long COVID symptoms
					Treatment type	
PA	M	65 +	White	Retired	12	Fatigue, shortness of breath, tight chest, brain fog, depression and anxiety
					Hospitalised/ICU	
PB	F	25–39	White	Professional	11	Fatigue, shortness of breath, tight chest, palpitations, headache, nausea, anxiety
					Hospitalised	
PC	F	18–24	Black	Student	10	Fatigue, dizziness, shortness of breath, changes in sense of smell and taste, depression
					Non-hospitalised	
PD	M	25–39	White	Professional	12	Fatigue, shortness of breath, tight chest, anxiety
					Hospitalised	
PE	F	25–39	White	Homemaker	12	Fatigue, brain fog, difficulty sleeping. depression
					Non-hospitalised	
PF	M	40–64	White	Professional	18	Fatigue, shortness of breath, brain fog, joint pain, depression
					Hospitalised	
PG	M	24–39	White	Professional	10	Fatigue, shortness of breath, brain fog, loss of appetite, depression and anxiety
					Hospitalised	
PH	F	40–64	Asian	Professional	18	Fatigue, brain fog, difficulty sleeping, depression
					Non-hospitalised	
PI	F	25–39	White	Professional	12	Fatigue, shortness of breath, brain fog, headache depression
					Non-hospitalised	
PJ	Male	40–64	White	Professional	12	Fatigue, brain fog, digestive problems, loss of appetite, dizziness, depression
					Non-hospitalised	
PK	Female	25–39	Black	Homemaker	18	Fatigue, loss of tase, loss of appetite, joint pain, depression, anxiety
					Non-hospitalised	
PL	F	25–39	White	Professional	24	Fatigue, brain fog, joint pains, poor appetite, diarrhoea, depression
					Non -hospitalised	
PM	F	40–64	Mixed	Professional	18	Fatigue, brain fog, skin rash, muscle aches, depression (frustration/anger)
					Hospitalised	
PN	F	18–24	Chinese	Student	10	Fatigue, brain fog, loss of taste, poor appetite, nausea, anxiety, depression
					Non-hospitalised	
PO	F	40–64	White	Volunteer	12	Fatigue, aches and pains, brain fog, sleep disturbance, depression (frustration/anger)
					Hospitalised	
PP	F	40–64	White	Professional	20	Fatigue, shortness of breath, aches and pains, depression (frustration/anger)
					Hospitalised	
PQ	F	18–24	Asian	Professional	24	Pins and needles, chills, rashes, joint pain, depression and anxiety
					Non-hospitalised	
PR	F	40–64	White	Professional	18	Fatigue, shortness of breath, difficulty sleeping, joint pain, depression
					Hospitalised	

### Interviews

Due to COVID-19 restrictions, the study took place remotely via an NHS secure Microsoft Teams system. There were two exceptions, where the interview was undertaken by telephone under participant request. Interviews took place over a period of four months between December 2021 and March 2022.

All participants were interviewed once individually, by the authors. All researchers received training in qualitative research, qualitative interviewing and Good Clinical Practice (GCP, NIHR). The researchers had also accessed specialist training in long COVID presentation in primary care. The method involved in-depth and semi-structured interviews by using an interview schedule as a topic guide and prompt sheet (Table 2).

**Table 2:** Overview of the semi-structured interview schedule

Area of interest	Example questions/prompts
Overview of long COVID lived experience	Can you summaries your overall experience of long COVID?
Diagnosis	Tell me about your experience of finding out you had long COVID?
Symptoms	What symptoms do you experience?
Impacts	How does long COVID affect your quality of life?
Support	What help have you received?
Management	How do you manage the illness?
Progression	Has your experience of living with long COVID changed over time?

The interview schedule was used help the participant into narrating a sufficient level of depth and detail. This allowed the participants to reflectively interpret the immensurable realities of their lived experience of long COVID illness. The average interview was 45 min, ranging from 35 to 90 min. There were no repeat or follow-up interviews. The interviews were audio-recorded on secure devices in line with participant consent. Contemporaneous notes were taken at each interview. Each interview was transcribed verbatim with all personal identifying information removed. Data were continually reviewed using a constant comparative approach throughout this process of data collection. As prompted by the topic guide, this was used to inform an iterative approach to the ongoing data collection and guiding subsequent interviews. Using this iterative approach, the primary author recruited until data saturation was attained [26]. Interviews continued until no more new themes emerged. At that point, the team determined that data saturation had been attained and the interviews were discontinued. The data saturation point was met with a sample size of 18 participants.

### Analysis

The IPA involved: An initial reading and re-reading the transcripts a number of times to obtain familiarity with the data and get a general sense of the accounts. In order to uncover the insider perspective into the lived experience of long COVID, a double hermeneutic approach was applied [24]. This occurred in two stages: the participant making sense of their personal and social lived experience of long COVD and the meaning they attach to it; the researcher making sense of the participant sense of living with long COVID. Using an inductive approach, initial codes were generated, and emergent themes were identified and explored. The coding process was data-driven, without preconceived ideas, hypotheses or attempts to integrate the data into pre-existing frameworks. Generated codes were refined as similar themes were clustered together and subthemes were identified which were then coalesced into major themes. Patterns and connections within and between transcripts were explored and integrated into the final analysis table. Final codes were organised in a thematic table. The initial analyses and coding were conducted by the primary author before being presented to the co-authors for review.

The researchers maintained a reflexive position throughout the analysis to minimise the risk of any presumptions that might affect the IPA findings. This research paper is written according to the Standards for Reporting Qualitative Research [27].

### Methodological considerations and reflexivity

Long COVID constitutes a rare illness needing further elucidation. In line with the aims and objectives of this study, IPA was considered suitable in gaining a rich insight into the phenomenological lived experiences of the participants.

Qualitative research is driven by trustworthiness which covers four criteria: credibility, dependability, confirmability, and transferability [28].

Credibility defines the truthfulness of the methodology including the data and analyses. In this study, credibility was strengthened by peer analysis and review by the researchers in order to attain consensus. This process was also useful in mitigating any potential for bias. Due to the sensitivity of the interview content, no member check was completed. However, the analyses were strengthened by the use of verbatim quotations from the participants.

Dependability defines the stability of the data over time. In this study, it was strengthened through continued interviewing until data saturation was attained. Interviews were also based on an interview schedule reviewed by expert patients. The same questions were asked to all participants and conducted by experienced researchers.

Confirmability refers to the neutrality of the data. In this study it was strengthened by the rigor in the conducted methodological processes including data collection and analysis. The methods are presented comprehensively in this paper.

Transferability defines whether the study can be generalized to groups beyond the participants. As this is a qualitative study, it does not claim wider generalizability. However, it included participants representing diverse ages, ethnicities, gender and class. Therefore, it can be transferred to wider groups. However, this study comprised of participants who had engaged with primary care services. Therefore, it does not claim generalizability to the wider community.

## Results

Each participant exhibited a subjective lived experience trajectory through their long COVID illness. The data analysis yielded four recurrent major themes, each with two to three subthemes in relation to the participant’s experience of long COVID: having an unknown chronic illness; living with uncertainty; regaining control; moving forward. These themes are discussed in detail below, with illustrative quotes from the participant interviews, and full summary in Table 3.

**Table 3:** Emerging themes and subthemes with illustrative quotes

Theme	Subtheme	Quotations
Having an unknown chronic illness	Reactions	1. It was very depressing at first when not much was known about it. You start to think it’s all in your head or you are going crazy. I joined support groups and forums. I also started searching the internet and medical journals. I was also talking to different sufferers and professionals (PD)
		2. It was very confusing. Nobody was telling me what it was even when I was not recovering for a long time. You don't know what's happening to you and nobody explained you anything, you feel left aside and like you're crazy, and nobody (PB) understands you, so yeah, it's not a very positive experience
		3. I thought I was imagining it because nobody understood. I mean like some of them were saying. Can't believe why you still moaning snap out of it and things like that It was only after going to the group and talking to other people that I related to their symptoms. I thought that is exactly how I feel, and my mind is not playing tricks on me (PR)
		4. I put two and two together because when I went to the doctors, they were dismissive because of my age group. They were paying more attention to my mum but for me they put it down to stress and said you will recover soon (PC)
		5. I felt dismissed by my doctors and started feeling like I was a nuisance because I kept pestering them and they did not believe me. I felt like no one around me understood. I had support around me. My family and friends were supportive, but I still felt that they did not understand, and I felt so alone (PQ)
		6. When it comes to doctors and nurses, I've got the utmost respect. They're doing a good job (PA)
	Denial	7. I am also resentful that I have not had support from work. A colleague was also misbelieving my symptoms and saying that once the infection is cleared then you are fine. There are other colleagues who have had COVID, and they are fine. It is almost like, what is wrong with you, why can’t you get over it. Why are you still suffering? If it was that simple, I would get over it because I do not want to be like this. If I could go back to the last day I felt physically well and prevent the COVID, I would. If I could go back to that day, maybe avoid COVID…(PM)
		8. I was feeling very depressed because I was feeling like a nobody was taking me seriously. And, yeah, my manager was like saying to me, how it's possible you are still like this? (PB)
		9. Talking to others who have been through similar is also helpful. I was talking to a colleague who also had COVID. She seemed to understand, and I did not need to explain myself too much (PP)
		10. My employer has not been supportive either… Yeah, it's just like they're like, oh, you are tired, but like, how can you be tired? (PE)
		11. At the start it was virtually impossible to accept it and. I pushed myself far too far and far. What was the most helpful was when they finally gave me the piece of paper that said. yes, you have long COVID because it then meant I could then look at the kind of the steps that others were taking (PF)
		12. I just felt like in this continual state of denial. Like, OK, you feel like you're going to get better and better, but you do know you're starting to get worse…I didn't realize that I was just pushing and pushing and pushing and this wasn't gonna get me anywhere. 'Cause nobody told me, you know, I mean, this was a few months in, but nobody knew enough about long COVID (PH)
	Physical and mental impacts	13. I have been having problems with the oxygen. I am on oxygen 24/7…They (consultant) did some tests and said to me, your lungs are better than they were last time…I still cannot walk more than 20 paces (PA)
		14. …I am just exhausted. I want to lie down, and I want to sleep but it is never enough…(PM)
		15. Because my physical health was bad it was affecting my mental health…There is a need to think about the mental health impacts (PG)
		16. My mental health is affected more than the actual physical problems (PJ)
		17. I have been having depression and I have never been depressed before…(PP)
		18. So basically, this long COVID has smashed my life into pieces. It's a whole mix of everything. The bad physical health means I am unable to do things, that gives me the anxiety which then effects my physical health. It all morphs into one (PF)
Living with uncertainty	Variable/unpredictable symptoms	19. I'd say you do get better, but I don't say that lightly because I appreciate that that long COVID is such a varied beast and It's multifaceted… (PI)
		20. I mean there are a lot of people with long COVID who have had significant organ damage… But there are a lot of us that physically appear to be OK. but still have huge amounts of inflammation, problems with fatigue, that just doesn't seem to be able to be explained by our traditional diagnosis methods. It is important to separate the two (PD)
	Frustration	21. I mean, I think if you wanted one word to actually sum up the whole long COVID experience, it is frustration…(PF)
		22. I was getting frustrated the symptoms seemed to alleviate just to come back again. It was affecting my daily activities (PD)
		23. It was just frustrating…You know? ‘Cause one minute I would be alright and then the next day that was it. I was extremely exhausted…absolutely exhausted. So, you know every day would be different (PR)
		24. Sometimes I feel like I can fight the world and other times I feel like I haven’t got the energy to do anything (P0)
		25. I just feel like I want to curl up into a ball because I can’t control when it happens (PM)
		26. It feels like your body has a mind of its own and is giving you mixed signals. My partner is being supportive. Yeah, and my friends as well, but they can only do so much, but they don't really get it to the point of what's going on in your body. It must also be frustrating for them, which I understand. (PF)
		27. I have a couple of friends who have been supportive. They have supported with food, shopping and bills until I got on my feet (PO)
		28. Work was supportive and reduced my hours…My daughter was very helpful and supportive. It’s like the roles were reversed. It is very difficult to accept yourself that you are not the same as you used to be and not being sure if this will change. It is very frustrating (PP)
	Loss of Identity/sense of purpose	29. When I was off work, I felt like I was just passing time with no purpose. Now I feel like the purpose is just my recovery that that's like the biggest purpose really (PG)
		30. I am a very proud man and when I couldn’t do basic things for myself it was difficult (PA)
		31. It feels like I am mourning the person I used to be. It feels like, when I had COVID, even though I did not die, a part of me did. It’s taken a part of my life with it… It’s almost like you are in a prison in your mind. Like you are there but just can’t get out. I feel like it all the time because I want to do things. I used to cook, do the shopping take care of the kids, it was all done. Now that does not happen. I would go to work take the kids out and still be ok. Now that is not possible… It does not affect just me. My children have also been affected. (PM)
		32. I could not do anything or play with the children. I was feeling lonely. I was frustrated (PK)
		33. It has broken me a lot and I have lost confidence…Life is for living but at the moment I feel like I am just existing. It has been like on a pause button (PJ)
		34. It kind of turned my world upside down. Actually, I would say it come. Because I particularly find it hard not to work because I've been very career focused for so long (PI)
		35. I felt like I had no like value in the family. And what was the point of it? You know, I had to really reassess. Like you know, what was my role in this family. I mean long COVID took all that away…(PH)
		36. It was affecting my relationships with other people (PN)
		37. Financially I am also affected…To be honest it is difficult to survive if I can’t work (PP)
Regaining control	Advocating for myself	38. Something inside me had a strong desire to get better. I was not getting the help, so it made me feel like I really had to advocate for myself, but like I felt good advocating for myself (PI)
		39. I told myself help was not coming and I had to do it myself. I became mindful of how I was using my energy and what I was doing. I had to reduce my levels of activity and then build them up again. I realised that I could do it and things started getting better. I set small goals and when I achieved the I improved. I also stopped putting pressure on myself (PL)
	Mastering the symptoms/self-management	40. I think the most helpful was the talking therapy. It was CBT and I really wanted to help myself. I was very happy with how I progressed through therapy and how it helped me (PN)
		41. I guess you just sort of get an idea of the pattern. Now I have figured out that the symptoms come in waves and are cyclical (PD)
		42. The symptoms are random but there is a build-up which I can feel coming on and I have had to adapt this (PL)
		43. Since I have long COVID I am unable to do the things I want to do. Then there is that battle… why can’t I do it? Then the body will kick in and say, this is why you can’t do it. I am just exhausted. I want to lie down, and I want to sleep but it is never enough. This is what I have learned you cannot sleep off fatigue because it is not the same as tiredness. You can sleep off tiredness but not fatigue (PM)
		44. I think I am now capable of pinpointing what I can do myself and when I need help. The symptoms are different for everyone. But I think it is really important to pinpoint the differences within yourself. I think a lot of people might not know that they are in long COVID. For example, me pinpointing that I was experiencing fatigue and noticing how it was having an impact on my life helped me realise the knock-on effect on my mental health. So, it is important to listen to your body and your mind (PN)
		45. I have learnt that young people can get long COVID too. A lot of young people may experience it but not know what it is. This is because it is not spoken about, and the focus is on older people. This can lead to it being missed because they might think it’s just stress or something (PC)
		46. I just didn't really understand. Kind of where it was coming from. And then I think it was at that point that I realized that you know this wasn't a normal recovery, you know. Yeah, so it wasn't going to be as straightforward a recovery as I thought it was going to be (PH)
		47. I have been pushing to find the strength inside myself. I am more careful with what I eat, and I have also been taking supplements and exercising so that I can be healthier and more energised. That is my way to help myself (PB)
Moving forward	Acceptance	48. I know that things will get better. A year ago, I thought I would never get better but now I am taking it day by day. I have developed compassion for myself and accepted what is going on (PL)
		49. I am a religious person and I think my faith in God has helped me. It made me feel that someone is helping me, and it helps me accept that what is meant to happen will happen and I believe that one should never give up (PP)
		50. The symptoms are still there but I am a lot happier now. My body is not 100%, and I don't think I will ever be 100% but I will try and be as healthy and as fit as possible (PR)
		51. Things have got better but I do often wonder if I will ever get back to what I used to be. It is taking such a long time and it does frustrate me sometimes. I have not come to terms with the long COVID yet. I would just like to get back to some of what I was like before (P0)
	Re-evaluation	52. I realise that the doctors are not bad people. They are trying to help but they do not have all the answers. But it does not mean that they are bad people. Also understanding that some treatments may not work for some people. It is about finding your own way and managing your expectations. In terms of power-balance, I would come in with a different expectation (PL)
		53. What I have learnt looking back now, before COVID, I thought I should never take a break from work or my routine. I almost thought that If I stopped then the world would stop. I now realise that the world does not stop if I stop, and that I need to take priorities and manage my time differently. I also need to accept myself as I am, and I am working on it (PP)
		54. I no longer place so much emphasis on work MM (PI)
		55. I don’t let my career define me (PH)
		56. I have changed my career aspirations and I would like to prioritise my health (PL)
		57. I mean, I consider myself very lucky to be alive…I try not to be hard on myself. I take each day one at a time. I used to worry about everything… I was always doing things for other people, it’s now time for me. I take my vitamin D tablet every day since the COVID. I also pamper myself a bit more like have me-time about once a week (P0)
		58. From like being young. I thought I was like untouchable, and I would never get ill, so it kind of taught me that we are all vulnerable in a way or health is valuable and we shouldn't take it for granted. My partner really stepped up to the plate. It meant a lot to me and made our relationship stronger…(PI)
		59. I have underestimated my own strength and I need to believe in myself a bit more (PL)
		60. Perhaps I am not as tough as I thought I was… I am very angry with some of my colleagues I thought were my friends…they have not been there for me (PJ)
	Re-defined sense of recovery	61. I feel like I am in a tunnel and that there will be light! When, I do not know….Hopefully, I am coming towards the end of the worst. I definitely know I am not at the beginning (PJ)
		62. …It was a comparison of how my body was feeling. For example, in January I thought I was better, but the fatigue was still there. In April, I noticed that the body was not as fatigued, and I was able to do things without losing too much energy. I think after I started noticing this, my mood started getting better… It was really surprising that depression and anxiety can exist in my life. I had never gone through such a long illness in my life and experienced symptoms that I never thought ever existed. The symptoms were very extreme for me. I am happy that I now understand my body better and it has prepared me for the future if I had to go through it again, I know how to deal with it or where to go to seek help. I now also know that I can help other people if they go through the same because I know what it feels like and what recommendations I can make for them (PN)
		63. Before I was tired everyday but now it happens every 2–3 days. Symptoms will come and go but I can cope… I am fine now (PK)
		64. I decided to focus on what I can do not what I cannot do. I took small career steps to start getting back to normality. And since doing that, I feel so much better. ‘Cause I just feel like I'm me again. It’s good. I feel like I'm back in control… I let go of being frustrated with my doctors (PQ)
		65. Right now, I am feeling better, and I do not experience the extreme symptoms of fatigue and dizziness like I was a few months ago… There is no cure right now, So I think the only way to help it a little bit is probably to build the mental strength to try and get yourself back up (PC)
		66. I have been slowly getting fitter. I used to do a lot of exercise. My goal is to get back to that level of fitness. I am not 100% yet but I know I can continue to expand my energy envelope. Being able to get to that level of fitness would signify recovery… Emotionally, I guess I'm a lot more upbeat (PD)
		67. Physically, I got there somehow and. I feel like I could be doing like exercise or everything again. I recovered from it, and, uh. No, I think I'm alright. I just need some professional to talk to me and just help me get through the mental side (PE)
		68. I know that essentially, long COVID is this kind of ongoing experiment sort of thing, and that because everyone is approaching it differently, it will be a while before we know the full picture. And so, my brain is kind of going well. If you get better quickly, then yeah, that's excellent! But because we don't know what the full picture is you know you can't expect to know a definitive timeline kind of thing. Because I’ll be disappointed with myself if I push myself too hard or set times. No one knows. Therefore, you get better when you get better (PF)

### Having an unknown chronic illness

This theme had three subthemes: *reactions*; *denial*; *physical and mental impacts*.

Having an unknown chronic illness evoked various reactions in the participants and those around them. The initial stages caused a range of feelings. For some this caused confusion, anxiety and/or depression (quotes 1, 2). For others there was denial (quotes 11, 12), and yet others were immediately mobilised to look for answers (quote 1). The reactions of others ranged from supportive (quotes 5, 9) to no support (quotes 3, 7, 8, 10). Some participants also reported feeling dismissed by healthcare professionals (quotes 4, 5), whilst others felt that they received the best care (quote 6). Most participants immediately recognised the correlated physical and mental health impacts of having an unknown chronic illness.

For example, PA, discussed the severity of his physiological sequelae. *‘I have been having problems with the oxygen. I am on oxygen 24/7…They (consultant) did some tests and said to me, your lungs are better than they were last time…I still cannot walk more than 20 paces.*’

PM expressed the severity of her fatigue symptoms…* ‘I am just exhausted. I want to lie down and I want to sleep but it’s never enough.’* PF was taken by the extreme interaction between the physical and mental health impacts. ‘*So basically, this long COVID has smashed my life into pieces. It's a whole mix of everything. The bad physical health means I am unable to do things, that gives me the anxiety which then affects my physical health. It all morphs into one’* (Table 1).

### Living with uncertainty

This theme had three subthemes: *Variable/unpredictable symptoms*; *frustration*; *loss of identity/sense of purpose*.

With the unknown trajectory of the illness came the uncertainty of living with it. All participants experienced symptoms which could not be anticipated, this ranged from variability (quotes 19–20) to unpredictability (quotes 24, 26). This uncertainty led to perceived stagnation and a sense of frustration for most participants (quotes 21, 22, 23, 25). For some, receiving support from others helped alleviate this sense of frustration (quotes 25, 26). For all participants living with uncertainty was coupled with a sense of loss of purpose or identity (quotes 29–37). For PA, this was expressed as a specific impact on his core-identity, ‘*I am a very proud man and when I couldn’t do basic things for myself it was difficult*’. For PM this was expressed in terms of the overall self and systemic identity and sense of purpose, ‘*It feels like I am mourning the person I used to be. It feels like, when I had COVID, even though I did not die, a part of me did. It’s taken a part of my life with it… It’s almost like you are in a prison in your mind. Like you are there but just can’t get out. I feel like it all the time because I want to do things. I used to cook, do the shopping take care of the kids, it was all done. Now that does not happen. I would go to work take the kids out and still be ok. Now that is not possible… It does not affect just me. My children have also been affected*’.

### Regaining control

This theme had two subthemes: *advocating for myself; mastering the symptoms/self-management.*

With the frustrations of uncertainty came the need to regain control. Most participants realised help was not coming and that they had to advocate for themselves (quotes 38, 39). With this realisation came the need to master the unknown symptoms. Most participants experientially learned the patterns in their symptoms and made the appropriate changes (quotes 41–47). Some also received supported guidance as part of their healthcare plan (quote 40). For example, PL expressed mastering his symptoms through personal experience, ‘*the symptoms are random but there is a build-up which I can feel coming on and I have had to adapt this*’. For PN, this was aided by therapy ‘*I think the most helpful was the talking therapy. It was CBT and I really wanted to help myself. I was very happy with how I progressed through therapy and how it helped me*’.

### Moving forward

This theme had three subthemes: *acceptance; re-evaluation; redefined sense of recovery.*

Once participants had gained a sense of mastery over their symptoms, they strengthened their locus of control to move forward. For most, this was marked by an acceptance of living with the chronic illness. This enabled most of the participants to recognise their progress (quotes 48–51). This was coupled with a new sense of instilled hope which enabled the participants to re-evaluate their subjective meanings of living with long COVID (quotes 52–60), and subjective sense of recovery and future coping (quotes 61–68). Overall, the participants reported a subjective sense of improved symptom severity and quality of life and outlook. Five out of the eighteen participants reported that they had made full recovery. The remaining thirteen participants still reported residual ongoing symptoms to varying degrees. Regardless of level of recovery, what transpired was the participants drive to move forward. For example, for PJ the symptoms were still present but he expressed his optimism, ‘*I feel like I am in a tunnel and that there will be light! When, I do not know…Hopefully, I am coming towards the end of the worst. I definitely know I am not at the beginning*’. PE acknowledged that her mental health impacts needed further attention, ‘*Physically, I got there somehow and. I feel like I could be doing like exercise or everything again. I recovered from it, and, uh. No, I think I'm alright. I just need some professional to talk to me and just help me get through the mental side*’. PF took the pragmatic approach, ‘*I know that essentially, long COVID is this kind of ongoing experiment sort of thing, and that because everyone is approaching it differently, it will be a while before we know the full picture. And so, my brain is kind of going well. If you get better quickly, then yeah, that's excellent! But because we don't know what the full picture is you know you can't expect to know a definitive timeline kind of thing. Because I’ll be disappointed with myself if I push myself too hard or set times. No one knows. Therefore, you get better when you get better*’.

## Discussion

Long COVID was coined and conceptualised by patients. This illness still remains unknown. Research to date highlights the enigma of long COVID illness and the individual differences in illness trajectories. In 2020, the National Institute for Health and Care Excellence (NICE) published guidelines on managing long COVID [29]. However, they acknowledged that there is much still unknown about this condition. A commentary published in the Lancet highlighted the need for long COVID guidelines to reflect the lived experience of sufferers [30]. The current study offers a response to this need in that it illustrates the phenomenological lived experience of long COVID.

### Relationship between the themes

The themes emerging from this study seem to show a sequential pattern. Overall, the participants in this study underwent the well-studied processes of integration in chronic illness, where they grieved the old-self and accepted/integrated the new-self. This sequential pattern is in line with the process of adjustment and integration [31]. Research suggests that the concept of integration is a central component to adjusting to chronic illness [31]. The current study adds further phenomenological information regarding patient experiences and overall illness trajectories. A key area to note is how the themes linked into one another and how they sometimes converged or diverged between participants, This highlights the idiosyncratic nature of long-COVID symptoms and the need to take an individualized person-centred approach to care as highlighted elsewhere e.g., [18].

### Comparison with existing literature

Few qualitative studies have been conducted to understand the perspective of sufferers of long-COVID. However, no in-depth analysis of the lived experiences and reflections of long-COVID patients has been conducted, A study by Wang et al. [32] used grounded theory and examined the perspectives of long-COVID from an online Q & A community of sufferers in China. One of their key findings was the public and self-stigma attached to long-COVID. Almgren et al. [33] used content analysis in a patient sample and found that they experienced uncertainty followed by new insights. Another study by Kingstone et al. [34] highlighted the importance of feeling understood and finding the right GP as important to long COVID patients. This notion was also highlighted in the current study. However, an additional notion was that some patients also expressed an empathy and gratitude for the help of their GPs and other healthcare providers.

Long-COVID as a chronic illness causes biographical disruption. According to the studies on rheumatoid arthritis, Bury [35] observed that having an unknown chronic illness leads to uncertainty in not knowing what will manifest. However, it has also been shown that this uncertainty leads to the mobilization of self-reclaiming resources. There seems to be a need to escape the sick role, although the mechanisms of this phenomenon remain unclear. As illustrated in the current study, the existing literature also highlights the common theme of long-COVID patents wanting to escape the sick role and reinforce their identity.

The current study derived the complex biopsychosocial individual, systemic and societal impacts of long COVID. On an individual level, this study highlighted the long-COVID disease trajectory from fear of uncertainty to taking control. This notion has also been pointed out by Engwall et al. [36]. On a systemic/societal level participants described the importance of having support and the helpfulness of having their symptoms validated by others including family, health professionals and employers. Participants also showed empathy towards other sufferers and the hard work of health professionals. Of note was the participants experiences in the work context. Some felt supported whereas others reported disappointment in the level of support from the workplace In their ‘return to work’ study, Kohn et al. [37] highlight the importance of raising employer awareness of the complications related to long-COVID.

The mental health impacts of long-COVID emerging in the current study have also been reported elsewhere e.g., the thematic analysis by Burton et al. [38]. However, the current study provides further new information regarding the participant’s lived experiences of how the physical health impacts interact with the mental health impact in a feedback-loop of negative-reinforcement.

There are still ongoing challenges in the diagnosis and management of long COVID [39]. As illustrated in the current study, using information from patients with a lived experience presents a rich avenue for research toto further unravel long COVID. This is particularly important as the current recommended care for long COVID is an integration of medical-care and self-management [29, 40]. The participants in this study highlighted the importance of interacting physical health and mental health impacts. This lends support to the NICE guidelines which have recommended integrated multidisciplinary team care models for managing long COVID patients [29].

### Limitations

As this is an epistemological rather than an empirical study, it does not claim generalisability to all long COVID sufferers. However, the small sample size, methodological rigor and iterative approach ensured trustworthiness of the findings. All participants in the current study were recruited from a single primary care mental health service and may not be representative of wider populations. Furthermore, although this study included diverse participant characteristics, it did not account for age, pre-long COVID health status or health inequalities which might impact the long COVID illness trajectories. This study also comprised of participants who had accessed mental health psychological support. It may be possible that these participants may present a better adjusted trajectory than those who chose not to take part or in the general population without psychological input.

## Conclusions

Findings from this study highlight significant features of long COVID disease from a patient phenomenological lived experience. The participants in this study captured the trajectory of long COVID from diagnosis, recovery and long-term management. There is still a lot to learn about long COVID illness. However, what is clear is that the patients are a rich source of data as they are the live actors who are intimately aware of what they are experiencing. Therefore, they can generate and illustrate live data which can be used to inform research and clinical practice. The identified biopsychosocial impacts of the long COVID lived experience highlight important areas for future research. For example, the current findings could be used to inform research on the pathophysiological mechanisms of long COVID. It could also inform research in understanding why not all patients make full physical recovery from long COVID. There is also a need for longitudinal randomized controlled trials to unravel the illness trajectories in various populations over time. Given the scarcity of lived experience research on long COVID and the rich findings from the current study, there is a need for more of this research. Participants provided feedback that they were willing to take part in long COVID research as part of their long COVID journey of helping themselves and others by contributing to clinically significant research. This further reiterates the importance of patient involvement.

## Data Availability

Original transcripts are available from the corresponding author on request.

## Abbreviations

COVID: Coronavirus disease
ICU: Intensive care unit
IPA: Interpretive phenomenological analysis
NHS: National Health Service
NICE: National Institute for Health and Care Excellence
NIHR: National Institute for Health and Care Research
